# Preparation and Characterization of Diene Rubbers/Silica Composites via Reactions of Hydroxyl Groups and Blocked Polyisocyanates

**DOI:** 10.3390/polym14030461

**Published:** 2022-01-24

**Authors:** Lun Ge, Qiang Liu

**Affiliations:** Key Laboratory of Rubber-Plastics of Ministry of Education, Qingdao University of Science & Technology, Qingdao 266042, China; 2019020002@mails.qust.edu.cn

**Keywords:** diene rubber, hydroxyl group, blocked polyisocyanates, crosslinking, silica

## Abstract

To improve the curing reaction rate and efficiency of sulfur-cured diene-based rubbers, the introduction of some chemical compounds as activators and accelerants is inevitably required, causing potential harm to humans and ecological systems. Moreover, silica is usually employed as a green filling material for rubber reinforcement, and a silane coupling agent is always required to improve its dispersion. Herein, we reported an effective method to cure hydroxyl-functionalized rubbers/silica composites with blocked polyisocyanates, avoiding the use of any other additives. The enhanced dispersion of silica by interaction with hydroxyl groups on molecular chains endowed the composites with high-mechanical performance. The mechanical properties and crosslinking kinetics of the resultant silica composites can be regulated by adjusting the content of hydroxyl groups in the rubber, as well as the amount of the blocked polyisocyanates. The dynamic heat build-up was related to the distance between crosslinking points. A SBROH/B-TDI/silica composite prepared with blocked toluene diisocyanatem (TDI) exhibited comparable tanδ (0.21 at 0 °C and 0.11 at 60 °C) to that of silica composites cured by sulfur with the help of a silane coupling agent (SBR/S/Si69/silica, 0.18 and 0.10), suggesting great applicable potential for new tire rubber compounds.

## 1. Introduction

Vulcanization involves crosslinking between polymer chains physically or chemically to generate three-dimensional network structures, which play a key role in achieving rubbery elasticity [1,2]. Even though various chemicals, such as peroxides [3], metal peroxides [4], amines [5] and resins [6], have been developed for the vulcanization of rubbers, the sulfur crosslinking system, first reported by Goodyear in 1839, is still most widely used in the industry. Due to the low crosslinking efficiency of using sulfur alone, activators and accelerants must be introduced to facilitate the crosslinking process and optimize the network performance [7,8]. However, these additives are usually toxic and will release harmful substances during the curing process [9]. For example, the activator zinc oxide is classified as toxic to aquatic organisms by the European Commission, and its application in rubber technology must be controlled by legislation [10]. Moreover, N-cyclohexyl-2-benzothi-azolesulphonamide (accelerant CZ), dibenzothiazoledisulfide (accelerant DM), and 2,2′-dibenzothiazole disulphide (accelerant MBTS) are classified as R43 (may cause sensitization by skin contact) and R50/53 (very toxic to aquatic organisms) according to the dangerous substances directive of European Union laws (67/548/EEC) [11]. Therefore, it is an urgent demand to explore an efficient and green method to crosslink diene rubbers. This leads to many attempts for developing new crosslinking structures, such as a physical crosslinking network formed by hydrogen bonds [12], ionic interaction [13], epoxy-functionalized elastomers crosslinked by dibasic acids [14,15], hydroxylated rubbers cured with various acrylates based on the oxa-Michael reaction [16], and diene rubbers crosslinked by the inverse vulcanized co-polymer [17].

In the early 1970s, a type of crosslinking system commonly referred to as urethane crosslinking was reported [18,19,20]. In this system, urea-urea crosslinks are created via a diisocyanate which is blocked by quione oxime, a tautomer of *p*-nitrosophenol. However, the high cost and poor compression preclude its widespread industrial applications. In 1990, Goodyear developed a crosslink technology based on reactivity rubbers (SBR or NBR) containing active hydrogens or blocked diisocyanates [21]. The resulting urethane crosslinks are remarkably durable under both thermal and hydrolytic conditions with carbon black (CB) as the filling material. However, the tensile strength of the prepared SBR/CB composites were quite low (about 12–18 MPa). In addition, the dynamic heat build-up study for this urethane crosslinking system was not involved.

Nanosilica is widely used as filler for “green tire” rubber matrix in tire industry [22,23], because it can reduce rolling resistance and enhance both mechanical properties and wet skid resistance [24,25]. The dispersion of filler is well known for deciding the performance of rubbers [26,27]. It is, however, difficult to disperse in non-polar rubber because there is a large number of silanol groups on the surface of silica [28,29]. A silane coupling agent is confirmed to be capable of improving the dispersion of silica in rubber significantly, but the need of additional high-temperature treatment increases the complexity of processing [30]. It has been very recently demonstrated that intra-chain functional groups in rubber can effectively improve the dispersion of silica through polar interaction or chemical connection [31,32,33].

Herein, we used a strategy to prepare hydroxyl-functionalized rubbers by epoxidation functionalization of diene rubbers with peroxide and subsequent acid ring opening reactions. Polyisocyanates are then used to crosslink hydroxyl-functionalized rubbers by using silica as a reinforcement material. We found that the hydroxyl groups in the rubber molecular chains can promote the dispersion of silica, thus resulting in high mechanical performance of hydroxyl-functionalized BR(BROH)/silica and hydroxyl-functionalized SBR(SBROH)/silica composites. In addition, the effect of hydroxyl groups content and chemical structure of blocked polyisocyanates on dynamic heat build-up for this urethane crosslinking system were studied.

## 2. Materials and Methods

### 2.1. Materials

The materials used were as follows: cis-1,4-polybutadiene rubber (BR, trade name: BR 9000, Beijing Yanshan Petrochemical Co. Ltd., Beijing, China); butadiene styrene rubber (SBR, trade name: SBR1502, styrene content of 23.5 wt%, Qilu Petrochemical Co. Ltd., Shandong, China); tetrahydrofuran (THF, Sinopharm Chemical Reagent Co. Ltd. Shanghai, China), 3-Chloroperbenzoic acid (mCPBA, 85%, Saan Chemical Technology Co. Ltd. Shanghai, China); hydrochloric acid (HCl, 38 wt% aqueous solution, Sinopharm Chemical Reagent Co. Ltd., Shanghai, China); hexamethylene diisocyanate, 1,4-Phenylene diisocyanate, and tolylene-2,4-diisocyanate (HDI, PPDI, TDI, Saan Chemical Technology Co. Ltd. Shanghai, China); butanone oxime (Saan Chemical Technology Co. Ltd. Shanghai, China); precipitated silica (trade name VN3, Evonik Degussa Co. Ltd. Shanghai, China); and blocked polyisocyanate (trade name VESTANAT B1358/100, referred to as BI, Evonik Degussa Co. Ltd. Shanghai, China). Other rubber additives were industrially available and used as received without further purification.

#### 2.1.1. Preparation of Hydroxylated BR and Hydroxylated SBR 

Pendant hydroxyl group functionalized BR (BROH_x_) and SBR (SBROH_x_) were synthesized according to the literature procedure [34]. Take BROH_3_ (containing 3 mol% hydroxyl groups) as an example; in a three-necked flask, 50 g of BR (0.926 mol C=C) were well dissolved in THF (0.05 g/mL). A solution of 5.68 g of mCPBA (0.028 mol) in 50 mL of THF was then added dropwise at room temperature (25 °C) into the polymer solution at such a rate that the mCPBA solution was added completely after 1 h. The reaction mixture was then further stirred for another 2 h. A solution of 4.7 mL of hydrochloric acid (0.056 mol of HCl) in 50 mL of THF was first prepared and transferred into a 100 mL dropping funnel; it was then added dropwise at room temperature (25 °C) into the epoxidized BR solution prepared previously. After the addition of the HCl solution, the reaction mixture was further stirred for another 2 h to complete the ring-opening reaction. The resultant solution was poured into H_2_O under stirring to precipitate the modified BR. The product was further dissolved in THF and precipitated by H_2_O repeatedly to fully remove the unreacted agents. Subsequently, the resulting polymer was dried in a vacuum oven at 40 °C to remove the residual solvent. The hydroxylated BR is denominated as BROH_x_, where x represents the molar percentage of the hydroxyl group. To investigate the effect of hydroxyl group content on cured rubber performance, BR with different contents of hydroxyl groups was synthesized according to the abovementioned procedures. Besides 3.0 mol%, other hydroxyl group contents were controlled to be 1.0 and 5.0 mol%. Additionally, SBR with different contents of hydroxyl groups (2.0, 5.0, and 7.0 mol%) were synthesized.

#### 2.1.2. Preparation of Blocked Polyisocyanates

In a three-port bottle equipped with magnetic stirring and a condensation tube and protected by dry nitrogen gas, 8.7 g TDI (0.05 mol), 10.4 g butanone oxime (0.12 mol), and 20 mL ethyl acetate were added, This mixture was reacted for 5 h at 77 °C and then distilled under reduced pressure to remove ethyl acetate. The resulting product was dried in a vacuum oven at 40 °C to remove the residual solvent. Blocked hexamethylene diisocyanate (BHDI); blocked 1,4-Phenylene diisocyanate (BPPDI); and blocked tolylene-2,4-diisocyanate (BTDI) were separately synthesized using butanone oxime as a blocking agent.

#### 2.1.3. Preparation of BROH_x_/BI/Silica and BR/S/Silica Composites

For the preparation of BROH_x_/BI/silica, 100 g of BROH_x_, 30 g of silica, and various amounts of BI (4, 6, 8 g) were mixed in a Haake internal mixer. The obtained compounds were then subjected to compression moulding at 160 °C for optimum time to produce BI-cured BROH_x_. The formed product was named BROH_x_BI_z_, where z is the content (phr, parts per hundreds of rubbers) of the crosslinker. Traditional sulfur-cured BR (BR/S) was prepared according to the abovementioned protocols based on the below formulation: BR 100 g; silica 30 g; bis[γ-(triethoxysilyl)propyl]tetrasulfide (Si69) 0 or 2.4 g; zinc oxide 5.0 g; stearic acid 1.0 g; N-Isopropyl-N′-phenyl-4-phenylenediamin (4010NA) 1.0 g; 1,3-Diphenylguanidine (D) 0.5 g; N-cyclohexyl-2-benzothiazole sulphonamide (CZ) 1.5 g; and sulfur 1.5 g.

#### 2.1.4. Preparation of SBROH_x_/BI/Silica and SBR/S/Silica Composites

For the preparation of SBROH_x_/BI/silica, 100 g of SBROH_x_; 50 g of silica; and blocked polyisocyanates (6 g BI, 3.06 g BHDI, 2.99 g BPPDI and 3.11 g BTDI, respectively) were mixed in a Haake internal mixer. The obtained compounds were then subjected to compression moulding at 160 °C for optimum time to produce BI-cured SBROH_x_. The formed product was named SBROH_x_BI_z_, where z is the content (phr, parts per hundreds of rubbers) of the crosslinker. Traditional sulfur-cured SBR (SBR/S) was prepared according to the abovementioned protocols based on the below formulation: SBR 100 g; silica 50 g; bis[γ-(triethoxysilyl)propyl]tetrasulfide (Si69) 0 or 5 g; zinc oxide 3.0 g; stearic acid 2.0 g; N-Isopropyl-N′-phenyl-4-phenylenediamin (4010NA) 1.0 g; N-tert-butylbenzothiazole-2-sulphenamide (NS) 2 g; and sulfur 1.5 g.

### 2.2. Methods

The ^1^H NMR spectra of products were recorded using a Bruker 500 spectrometer with CDCl_3_ as a solvent and tetramethylsilane (TMS) as an internal standard. A Fourier-transform infrared spectroscopy (FTIR) analysis was conducted on a VERTEX 70 instrument in attenuated total reflectance (ATR) mode. The spectra were collected over the wavenumber of 4000−500 cm^−1^ with a 4 cm^−1^ resolution and 32 scans. The molecular weight (*M*_n_) and molecular weight distribution (PDI, *M*_w_/*M*_n_) of the polymers were measured by GPC equipped with an RI detector (GPC-RI) and an EcoSEC832 pump. The flow rate of tetrahydrofuran (THF) was 0.35 mL/min. Differential scanning calorimetry (DSC) was recorded on a DSC204F1 instrument (NETZSCH) and thermograms were recorded using a heating rate of 10 °C/min under nitrogen atmosphere from −80 °C to 40 °C. Crosslinking kinetics were determined at different temperatures on an MDR2000 (Alpha Technologies, Wilmington, DE, USA) vulcameter. The tensile and tear test was carried out by a Zwick/Roell Z005 instrument. Shore A hardness was performed on a durometer (GOTECH Testing Machines Company). Resilience was performed on a rubber rebound testing machine (GOTECH Testing Machines Company). Compression set retention rate was performed on a rubber compression permanent deformer at 25% compression for 72 h at room temperature. Crosslinking density was measured by the equilibrium swelling experiment (see Supporting Information). Dynamic mechanical analysis (DMA) was performed using a NETZSCH EPlexor 500N analyzer under the conditions of a frequency of 10 Hz at the speed of 3 °C/min from −120 °C to 80 °C. Filler networks were analyzed using an RPA 2000 at 60 °C (Alpha Technologies, Wilmington, DE, USA). The compounds were analyzed over the strain range of 0.28–400% at 1 Hz. Transmission electron microscopy (TEM) for the ultramicrotomed samples was performed on a JEM-2100 instrument (Tokyo, Japan).

## 3. Results

### 3.1. Hydroxyl Functionalization of Butadiene Rubber

**Scheme 1:**
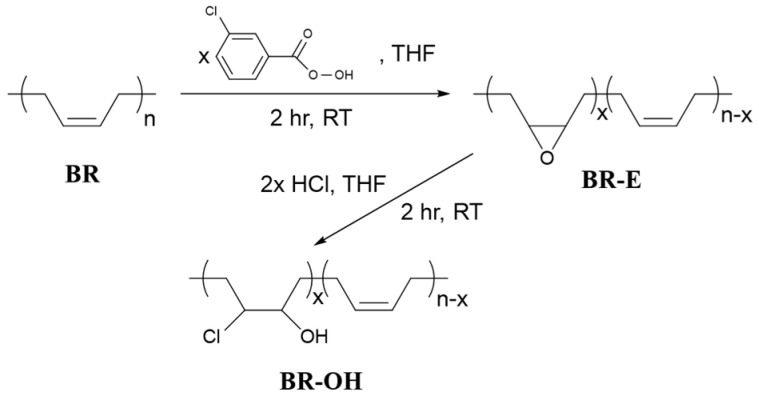
Preparation of hydroxyl-functionalized rubbers.

A series of pendant hydroxyl-group-functionalized *cis*-polybutadiene rubber (denominated as BROH_x_, where x represents the molar ratio of structural units containing hydroxyl groups) were synthesized according to Scheme 1. The commercial blocked polyisocyanates, i.e., B1358 (a closed ring aliphatic polyisocyanate that is based on the isophorone diisocyanate), referred to as BI, was used to cure the silica-filled hydroxyl functionalized rubbers. The formed product was named BROH_x_BI_z_, where z is the content (phr, parts per hundreds of rubbers) of the crosslinker.

**Figure 1:**
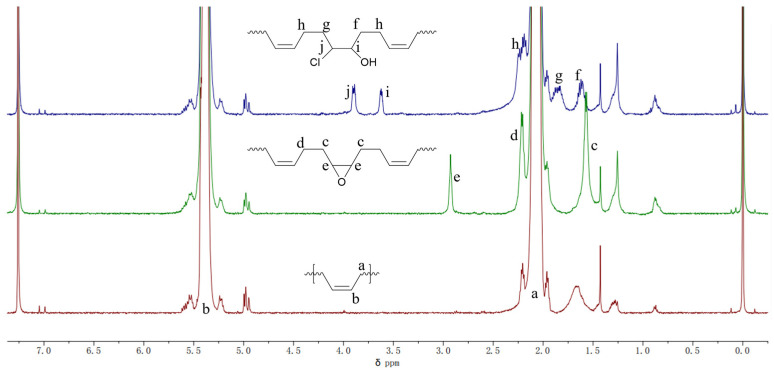
^1^H NMR spectra of BR with a 3 mol % degree of modification.

The chemical structures of formed products were investigated by FTIR spectra (Figure S1). The peak transmittance intensity was normalized to the peak at around 3008 cm^−1^ ascribed to C−H bending vibration and used to compare the changes of each moiety. The intensity of peaks around 3575 cm^−1^ corresponding to the O−H bending increased continuously while the peak intensity of around 2939 cm^−1^ representing =C−H weakened with the increasing amount of *m*-chloroperoxybenzoic acid (mCPBA) and hydrochloric acid, indicating that the hydroxyl group content increased in the polybutadiene rubber (BR). The hydroxyl group contents were quantitatively determined by ^1^H NMR spectra as listed in Table S1. The peak at 2.74 ppm (Figure 1) assigned to epoxide groups, which was formed by the epoxidation of mCPBA, completely disappeared after the ring opening reaction in the presence of hydrochloric acid. The double peaks at 3.73 and 3.96 ppm corresponding to hydroxyl and chlorine groups were used to calculate the efficiency of ring opening reactions with epoxide and chlorine, respectively [34]. As shown in Table S1, both efficiencies were very high. The molecular weight (*M*_W_) and molecular weight distribution (MWD) of BROH_x_ before and after hydroxylation were determined by GPC (Figure S2 and Table S2). The *M*_W_ slightly increased as the content of hydroxyl groups increased, indicating the successful grafting of hydroxyl groups onto BR chains.

### 3.2. Crosslinking of BROH_x_ with Blocked Polyisocyanates

**Figure 2:**
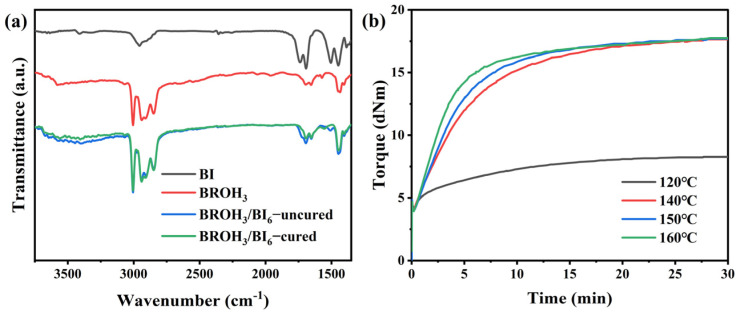
(**a**) FTIR spectra of BI, BROH_3_, and BROH_3_/BI compounds and BROH_3_/BI_6_ vulcanizate and (**b**) curing profiles of BROH_3_/BI/silica composites at different curing temperatures.

**Scheme 2:**
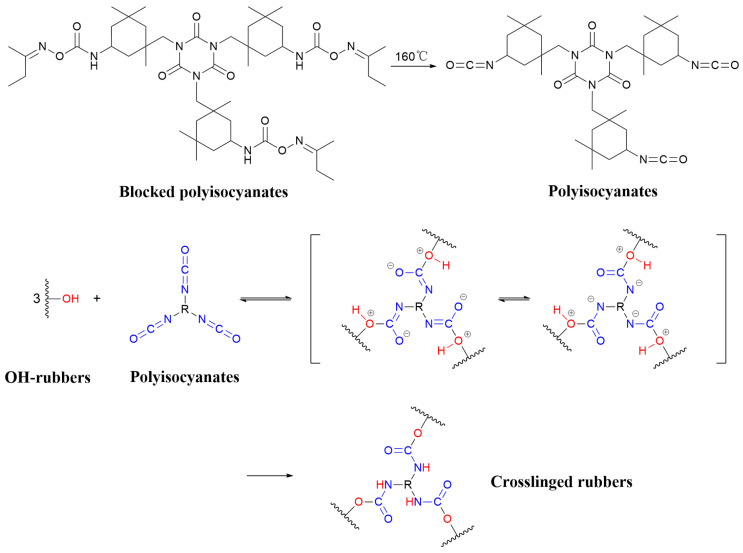
The unblocking of blocked polyisocyanates and the vulcanization mechanism of polyisocyanates-cured BROH_x_/BI/silica composite.

To explore the reaction mechanism between BROH_x_ and blocked polyisocyanates, we monitored the FTIR spectra of BI, BROH_3_, and BROH_3_/BI_6_ compounds and BROH_3_/BI_6_ vulcanizate. As seen from Figure 2a, the intensity of peaks at 1739 cm^−1^ related to C=O stretching vibration in blocked polyisocyanates decreases after crosslinking, indicating the successful unblocking of blocked polyisocyanates [35]. Upon the addition of silica, the degree of association of hydroxyl groups increases, leading to peak broadening and red shift to a lower wavenumber. The decreased intensity of peaks at 3440 cm^−1^ assigned to hydroxyl groups after the curing process confirms clearly the partial consumption of hydroxyl groups in BROH_3_ by unblocked isocyanate groups. The curing behavior of the BROH_3_/BI/silica composite was examined to verify the effectiveness of crosslinking reactions. Figure 2b showed the curing profiles of BROH_3_/BI/silica at different temperatures. At temperatures above the unblocking temperature (130 °C) of BI, the curing process was accelerated as the curing temperature increased, and the maximum torques (MH) were nearly the same, indicating the completion of crosslinking reactions between newly released highly active isocyanate groups and hydroxyl groups. Additionally, the curing curve began to level off after 15 min at temperatures higher than 150 °C, similar to traditional sulphur-based curing systems. However, at temperatures below the unblocking temperature, the crosslinking reaction was rather slow since the reaction can only rely on a trace amount of free isocyanate groups. For the same reason, the low temperature blending process did not cause early crosslinking and can ensure the processing safely. The crosslinking chemistry based on BROH_x_ and blocked polyisocyanates was summarized in Scheme 2.

### 3.3. Mechanical Properties of BROH_x_/BI/Silica Composites

**Figure 3:**
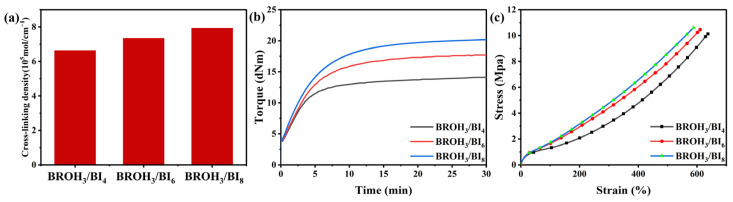
(**a**) crosslink density, (**b**) curing curves, and (**c**) stress−strain curves of BROH_3_ cured by different amounts of BI.

The mechanical properties of formed rubber composites can be manipulated by adjusting the BI content. Figure 3a was the crosslinking density plot of BROH_x_/BI/silica composite vs. BI amount. The crosslinking density increased gradually with the increase in BI amount due to the increase in reaction points. With the increase in BI dosage from 4 to 8 phr, the crosslinking density enhanced from 6.63 to 8 ×10^−5^ mol/cm^3^. Figure 3b presents the curing curves of BROH_3_ using different BI doses. With the increase in BI content, the crosslinking density and MH value increased monotonously. Moreover, the crosslinker content had little effect on the curing time. The stress−strain curves of BROH_x_/BI/silica composites areshown in Figure 3c. With the increase in BI amount, the tensile strength and modulus of BROH_x_/BI/silica composites increased obviously, and the breaking elongation decreased because of increasing crosslinking density.

**Figure 4:**
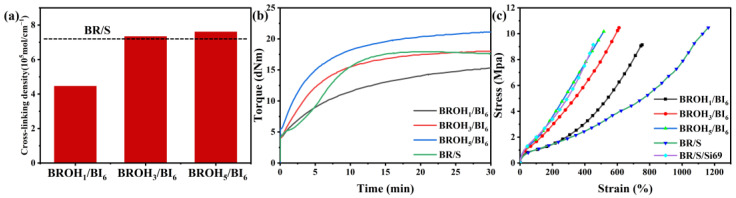
(**a**) crosslink density, (**b**) curing curves, and (**c**) stress−strain curves of BROH_x_ cured by 6 phr BI.

The effect of hydroxyl group content on the performance of cured rubber was further investigated. As shown in Figure 4a, at a constant dosage of BI (6 phr), the crosslinking density of BROH_x_ increased with the increasing hydroxyl group content because of increasing amount of curing sites. The crosslinking density exhibited an abrupt increase from 4.46 to 7.34 × 10^−5^ mol/cm^3^ when the hydroxyl group content increased from 1 to 3 mol%. In contrast, a further increase in the hydroxyl content from 3 to 5 mol%, only led to a slight increase in crosslinking density. The possible reason was that effective crosslinking can only be realized when at least two of the three isocyanate groups in each BI react with the rubber chains. When the BROH_x_ hydroxyl content was low (e.g., BROH_1_), no effective crosslinking could be formed because of the reduced possibility of the limited hydroxyl groups to react with multiple isocyanate groups in each BI. At a higher content of hydroxyl groups (e.g., BROH_5_), more hydroxyl groups were accessible to isocyanate groups of BI for effective crosslinking. Figure 4b depicts the curing curves of BROH_x_/BI/silica composites with the hydroxyl group contents. High hydroxyl group content led to a shortened curing time and an increased MH due to a higher crosslinking density. The stress−strain curves of BROH_x_/BI/silica composites are shown in Figure 4c. Both the tensile strength and modulus were enhanced with the increase in hydroxyl group content. The elongation at break depended also remarkably on the hydroxyl content, which was attributed to the increment of the crosslinking density, as well.

Notably, the modulus of the BROH_3_/BI_6_/silica composite was as high as 4.4 MPa at a strain of 300%, close to the value of the BR silica composite with Si69 added (Table 1, BR/S/Si69/silica), much higher than those of BR composites with similar crosslinking density prepared using the traditional sulphur-cured BR/S/silica composite (Figure 4a, BR/S/silica). Additionally, the resilience and the permanent deformation retention in compression were all enhanced with the increase in hydroxyl content. The tensile strength and modulus of BROH_5_/BI_6_ were even superior to the traditional BR/S/Si69/silica composite, but the values of permanent set, resilience, and compression set were higher than that of the BR/S/Si69 composite at different hydroxyl contents.

### 3.4. Mechanical Properties of SBROH_x_/BI/Silica Composites

**Figure 5:**
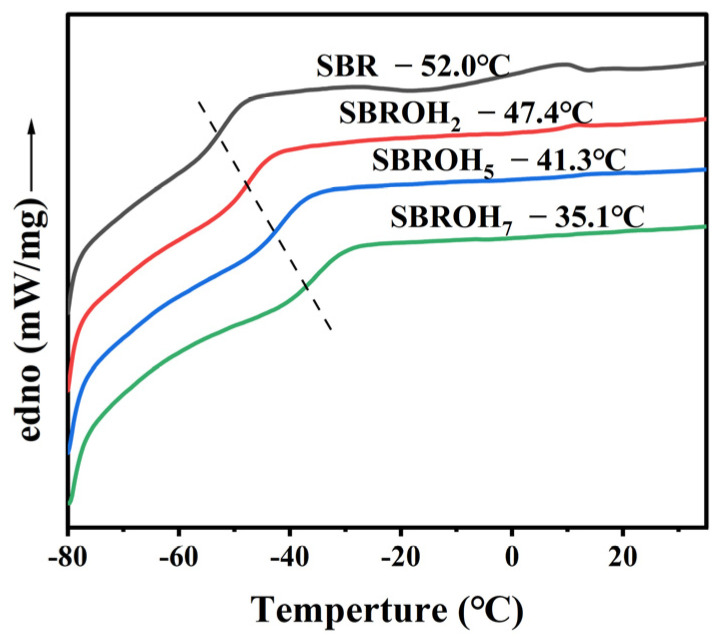
DSC curves of SBROH_x_.

It should be noted that this BI-cured system was generally applicable to other diene-based rubbers, such as styrene butadiene rubber (SBR). A series of pendant hydroxyl-group-functionalized SBR (denominated as SBROH_x_, FTIR, ^1^H NMR, and GPC, which are shown in Figures S3 and S4 and Table S3) were synthesized in the same way. Additionally, the effect of hydroxyl content on glass transition temperature (*T*_g_) was characterized by a DSC test. As shown in Figure 5, with the increase in content of hydroxyl groups, *T*_g_ gradually increased from −52 to −35.1 °C, which may be attributed to the enhancement of chain polarity and hydrogen bonding interactions. There were some changes in the DSC curve of SBR at about −20 and 10 °C. It is proposed that these changes may be attributed to some unknown thermodynamic transformations originating from the impurities and additives in commercial SBR.

**Figure 6:**
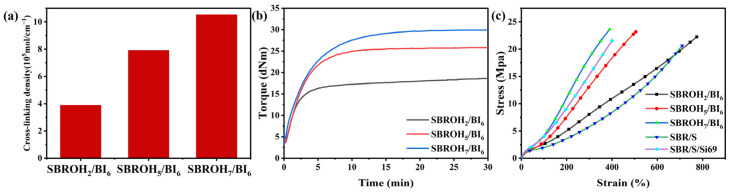
(**a**) crosslink density, (**b**) curing curves, and (**c**) stress−strain curves of SBROH_x_ cured by 6 phr BI.

The effect of hydroxyl content on the properties of SBROH_x_/BI/silica composites was investigated with a fixed dosage (6 phr) of BI. As shown in Figure 6, as with BROHx/BI/Silica composites, the crosslinking density increased with the increase in hydroxyl content. The MH also increased with the increase in crosslinking density. As shown in Figure 6c, the elongation at break of SBROH_2_ was close to that of the SBR/S/silica composite (cured by sulfur), while the modulus and tensile strength were higher. The modulus for SBROH_x_ can be further improved close to or even higher than that of the SBR/S/Si69/silica composite (cured by sulfur with the help of Si69). However, the elongation at break would be decreased with contents of hydroxyl groups (see Table 2).

### 3.5. Dispersion of Silica in the Rubber Matrix

**Figure 7:**
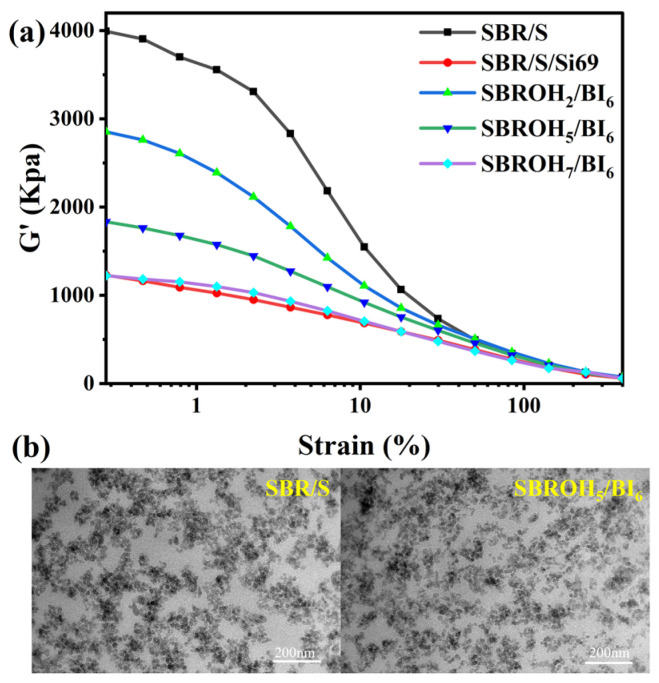
(**a**) RPA analysis, (**b**) TEM images of SBR/silica and SBROH/silica composites.

We recall that the dispersion of filler is key for deciding the performance of rubber [26,27]. It was found that the introduction of hydroxyl groups facilitates the dispersion of silica (TEM image Figure 7b) because of the enhanced interaction of rubber chains with silica. An RPA analysis was performed on rubbers before and after hydroxylation treatment. As shown in Figure 7a, typical Payne effects were observed for SBR/S/silica composites: the initial very high modulus decreases rapidly with the increase in strain [36]. In contrast, the SBROH/BI/silica composites exhibited a much lower initial storage modulus, and the Payne effects were weaker with higher hydroxyl contents, even equivalent to that of te SBR/S/Si69/Silica composite at a hydroxyl content of 7 mol%, suggesting a good dispersion of silica. Similarly, Payne effects with BROH/BI/Silica composites (Figure S5) were weakened significantly with the increasing content of hydroxyl groups.

### 3.6. Dynamic Mechanical Properties of SBROH_5_/Silica Composites Cured with Different Polyisocyanates

**Figure 8:**
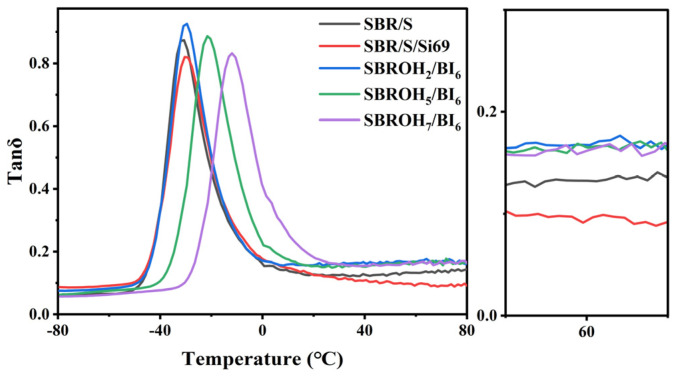
Tanδ-T curves of SBR/S/silica, SBR/S/Si69/silica, and SBROH_x_/silica composites.

A trade-off was the decrease in resilience and the permanent deformation retention in compression for BROH/BI/silica composites and SBROH/BI/silica composites (see Table 1 and Table 2). The effect of hydroxyl groups content on the dynamic behavior of the SBROH/BI/silica composites is shown in Figure 8. The *T*_g_ increased with the increase in hydroxyl groups content, coherent with the observation in DSC tests. However, compared to the SBR/S/Si69/silica composite, the tanδ at 60 °C for SBROH/BI/silica composites were all clearly higher, which is possibly because of a bigger inter crosslink distance. Although some damping materials required higher tanδ, low tanδ and dynamic heat build-up is more desirable for more applications, such as tread compound. Therefore, the effect of the isocyanate structure was further studied by varying the distance between crosslinking points.

**Scheme 3:**
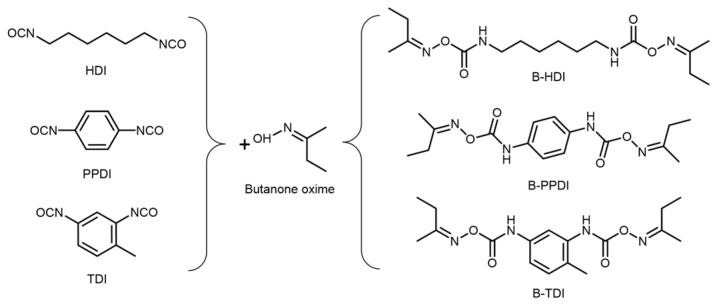
Preparation of different blocked diisocyanates.

Blocked hexamethylene diisocyanate (B-HDI); 1,4-Phenylene diisocyanate (B-PPDI); and toluene-2,4-diisocyanate (B-TDI) were synthesized (Scheme 3) using butanone oxime. The distance between the two NCO groups for these three diisocyanates increased sequentially. The blocking reaction was complete, as the peak at 2270 cm^−1^ assigned to NCO groups in TDI disappeared after the reaction (FTIR spectra, Figure S6).

**Figure 9:**
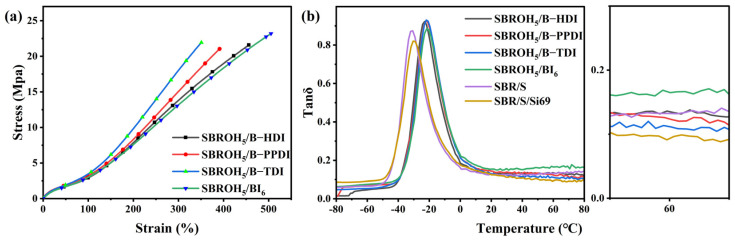
(**a**) stress−strain curves and (**b**) tanδ-T curves of SBROHx cured by different blocked diisocyanates.

Firstly, the SBROH_5_/Silica composites were vulcanized under the same molar ratio of NCO/OH (6 phr BI, 3.06 phr BHDI, 2.99 phr BPPDI, and 3.11 phr BTDI). The curing curves are shown in Figure S7, and all three samples were cured effectively. As shown in Figure 9a, the tensile strengths for these three blocked diisocyanates were similar, but the modulus increased gradually in the order of B-HDI, B-PPDI, and B-TDI (Table 3). Furthermore, as shown in tanδ-T curves (Figure 9b): (1) the structure of blocked diisocyanate had little effect on *T*_g_; (2) surprisingly, tanδ values at 60 °C for B-HDI, B-PPDI, and B-TDI were all significantly smaller than that of BI and decreased in the order of HDI, PPDI, and TDI. Therefore, the dynamic heat build-up was relevant to the chemical structures of diisocyanates. The tanδ values with TDI were close to those of the SBR/S/Si69/silica and SBROH_x_/silica composites, as shown in Table 4. Moreover, the resilience properties increased, and the compression set decreased, superior to the SBR/S/Si69/silica composite. It also should be noted that the higher tanδ values at 0 °C (0.21) (Table 4) suggested improved wet skid resistance in tire compound application.

## 4. Conclusions

In summary, high crosslinking efficiency can be achieved without the introduction of other toxic activators and accelerants via sulfurization chemistry based on the reaction of hydroxyl groups with blocked polyisocyanates. In addition, the hydroxyl groups in rubber chains promoted the dispersion of silica filler. A good dispersion of silica was attained even without the usage of the silane coupling agent. The mechanical properties and crosslinking density of the samples can be readily adjusted by varying the content of hydroxyl groups and the amount of blocked polyisocyanates. Compared to the sulphur-cured counterpart of the SBR/S/Si69/silica composite with a similar crosslinking density, the blocked polyisocyanates-cured SBROH_5_/silica composite exhibited a higher tensile modulus. In addition, the dynamic heat build-up was affected by the distance between the two NCO groups in the diisocyanate. The SBROH_5_/B-TDI/silica composite showed superior resilience properties and compression set, as well as a low tanδ value at 0 °C, which was very close to that of the SBR/S/Si69/silica composite. Overall, this work provides a new and efficient pathway to fabricate rubbers with novel structures and high mechanical and dynamic heat build-up properties. Many other properties and the applications for this strategy are currently being explored in our laboratory.

## Figures and Tables

**Table 1:** Mechanical properties of BROH/BI/silica composites.

Sample	Tensile Strength (MPa)	Modulus at 100% (MPa)	Modulus at 300% (MPa)	Elongation at Break (%)	Shore A Hardness	Permanent Set (%)	Resilience (%)	Compression Set (%)
BR/S	10.4	1.0	1.9	1101	60	24	62.8	21.3
BR/S/Si69	9.1	1.9	5.1	451	66	5	79.4	6.4
BROH_1_/BI_6_	9.1	1.0	2.1	754	58	22	61.4	25.7
BROH_3_/BI_6_	10.5	1.7	4.4	610	60	16	64.3	21.8
BROH_5_/BI_6_	10.1	1.9	5.6	517	63	12	68.8	14.9

**Table 2:** Mechanical properties of SBROH/BI/silica composites.

Sample	Tensile Strength (MPa)	Modulus at 100% (MPa)	Modulus at 300% (MPa)	Elongation at Break (%)	Shore A Hardness	Permanent Set (%)	Resilience (%)	Compression Set (%)
SBR/S	20.8	2.0	5.8	709	70	30	44.6	21.3
SBR/S/Si69	21.6	4.0	15.3	401	76	9	51.4	11.1
SBROH_2_/BI_6_	22.2	2.6	8.0	774	72	22	42.6	29.8
SBROH_5_/BI_6_	23.2	3.0	13.2	505	76	15	44.2	23.1
SBROH_7_/BI_6_	23.6	4.22	18.5	389	80	10	47.3	20.2

**Table 3:** Mechanical properties of different blocked polyisocyanates-cured SBROH_5_ composites.

Sample	Tensile Strength (MPa)	Modulus at 100% (MPa)	Modulus at 300% (MPa)	Elongation at Break (%)	Shore A Hardness	Permanent Set (%)	Resilience (%)	Compression Set (%)
SBROH_5_/B-HDI	21.6	2.9	13.8	456	77	8	52.1	10.3
SBROH_5_/B-PPDI	21.0	3.2	15.0	391	76	8	52.9	8.9
SBROH_5_/B-TDI	21.9	3.5	18.2	351	77	5	53.2	8.3

**Table 4:** Tanδ at 0 and 60 °C of composites.

Sample	Tanδ at 0 °C	Tanδ at 60 °C
SBR/S	0.16	0.13
SBR/S/Si69	0.18	0.10
SBROH_2_/BI_6_	0.17	0.17
SBROH_5_/BI_6_	0.22	0.16
SBROH_7_/BI_6_	0.41	0.16
SBROH_5_/B-HDI	0.19	0.13
SBROH_5_/B-PPDI	0.21	0.12
SBROH_5_/B-TDI	0.21	0.11

## Data Availability

The data presented in this study are available on request from the corresponding author.

## Supplementary Materials

The following supporting information can be downloaded at https://www.mdpi.com/article/10.3390/polym14030461/s1: Crosslinking density; Figure S1: Evolution of FTIR spectra of BROH_x_ with increasing mCPBA and HCl dose; Figure S2: The molecular weight distribution (MWD) of BROH_x_; Figure S3: Evolution of FTIR spectra of SBROH_x_; Figure S4: ^1^H NMR spectra of SBROH_5_; Figure S5: RPA curves of BROH_x_ cured by 6 phr BI; Figure S6: Evolution of FTIR spectra of TDI and blocked TDI; Figure S7: Curing curves of SBROH_5_ cured by different blocked diisocyanates; Table S1: Degree of hydroxylation under different conditions; Table S2: Molecular weight distribution of BR after hydroxylation; Table S3: Molecular weight distribution of SBR after hydroxylation.
