# Supplementary material for: Preparation and Characterization of Diene Rubbers/Silica Composites via Reactions of Hydroxyl Groups and Blocked Polyisocyanates
Source: Polymers (Basel). 2022 Jan 24;14(3):461. doi: 10.3390/polym14030461 (PMC8839315; doi:10.3390/polym14030461)
Supplement: Supplementary file 1 [file polymers-14-00461-s001.zip › Supplementary Materials(1).pdf]

polymers

MDPI

Supplementary Materials

Preparation and Characterization of Diene Rubbers/Silica Composites via Reactions of Hydroxyl Groups and Blocked Polyisocyanates

Lun Ge [1] and Qiang Liu [1,*]

[1] Key Laboratory of Rubber-Plastics of Ministry of Education, Qingdao University of Science & Technology, Qingdao 266042, Shandong, China
* Correspondence: liuqiang@qust.edu.cn

Cross-linking density. Cross-linking density was measured by the equilibrium swelling experiment [1]. The vulcanizates were immersed in toluene at room temperature for 72 h. Subsequently, the samples are removed from the solvent and immediately weighed after wiping off the surface toluene. The samples were further dried in a vacuum oven at 60 °C until constant weight. The cross-linking density was determined as the classical Flory-Rehner equation [2].

$$V_e = \frac{\ln(1 - V_r) + V_r + \chi {V_r}^2}{V_s({V_r}^{1/3} - 2V_r/f)}$$

where V_r is the volume fraction of rubber in the swollen gel, V_s is the molar volume of the solvent (106.5 cm^3/mol for toluene), and χ is the Flory-Huggins solvent-polymer interaction parameter ($\chi_{BR\text{-}toluene}$ is 0.34 and $\chi_{SBR\text{-}toluene}$ is 0.413) [3,4]. f is the functionality of interlinkage, which is 4 for sulfur cured non-end crosslinked diene rubbers while it is 6 for BI and 4 for B-HDI, B-PPDI, B-TDI used in this paper [5]. V_r was calculated according to the following equation:

$$V_r = \frac{(m_2 - m_0\varphi)/\rho_r}{(m_2 - m_0\varphi)/\rho_r + (m_1 - m_2)/\rho_s}$$

where φ is the weight fraction of the insoluble components (such as silica), and ϱr and ϱs are the density of rubber and solvent, respectively.

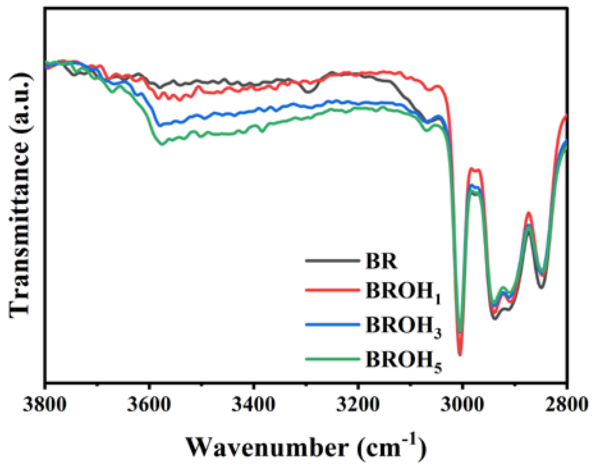

Figure S1. Evolution of FTIR spectra of $BROH_x$ with increasing mCPBA and HCl dose.

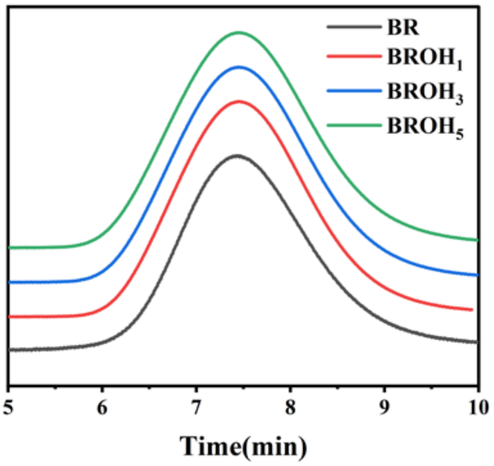

Figure S2. The molecular weight distribution (MWD) of BROHx.

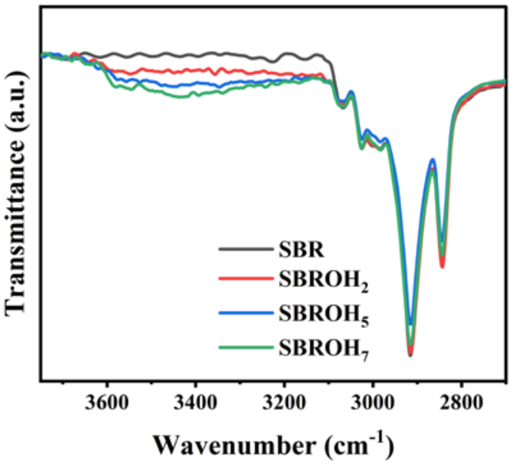

Figure S3. Evolution of FTIR spectra of SBROHx.

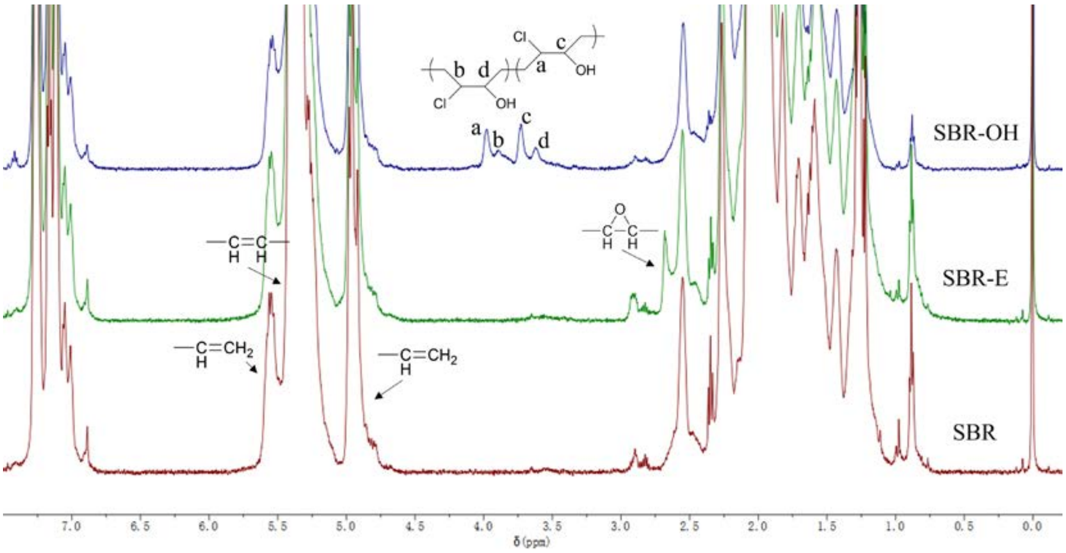

Figure S4. ^{1}H NMR spectra of SBROH5.

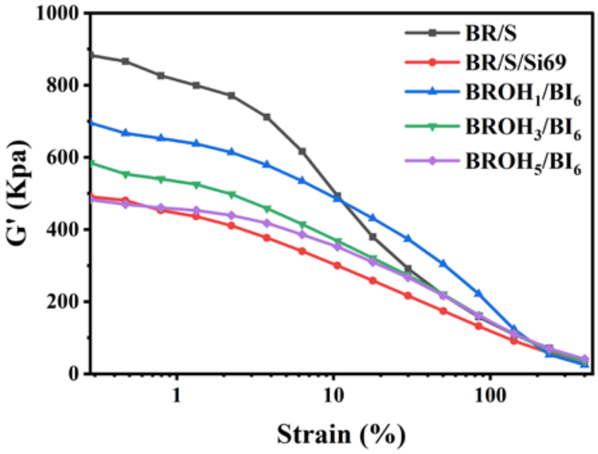

Figure S5. RPA curves of $BROH_x$ cured by 6 phr BI.

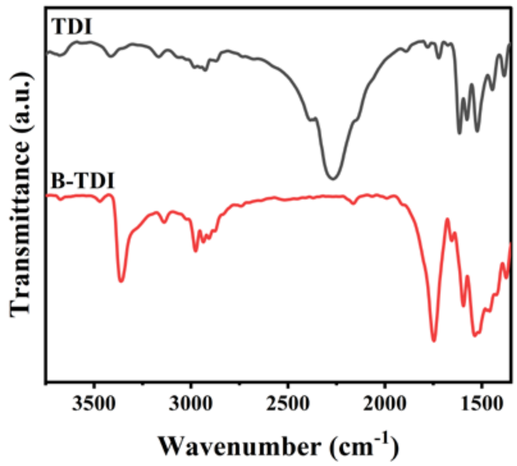

Figure S6. Evolution of FTIR spectra of TDI and blocked TDI.

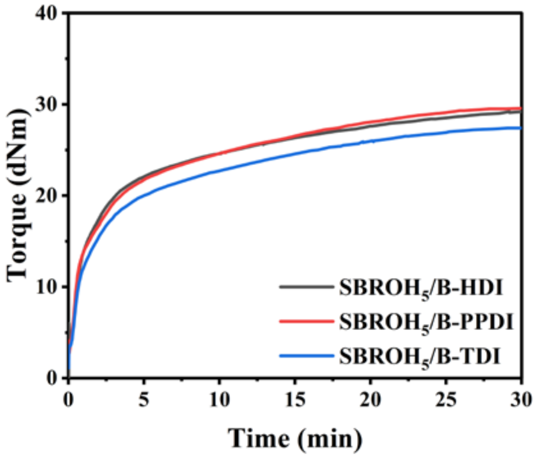

Figure S7. Curing curves of $SBROH_5$ cured by different blocked diisocyanates.

Table. S1. Degree of hydroxylation under different conditions.

Sample	mCPBA(g)	HCl(ml)	deg of hydroxylation (mol %)
$BROH_1$	1.89	1.6	0.99
$BROH_3$	5.68	4.7	2.96
$BROH_5$	9.47	7.8	4.87

Table. S2. Molecular weight distribution of BR after hydroxylation.

Sample	*Mn*	*Mw*	MWD
BR	126300	493500	3.9
$BROH_1$	128400	522700	4.1
$BROH_3$	131100	549800	4.2
$BROH_5$	134000	577500	4.3

Table. S3. Molecular weight distribution of SBR after hydroxylation.

Sample	*Mn*	*Mw*	MWD
SBR	78300	733200	9.4
$SBROH_2$	80800	807100	9.9
$SBROH_5$	83300	853400	10.2
$SBROH_7$	84400	879200	10.4

References

1. Guo, B.; Chen, F.; Lei, Y.; Liu, X.; Wan, J.; Jia, D. Styrene-butadiene rubber/halloysite nanotubes nanocomposites modified by sorbic acid. *Appl. Surf. Sci.* **2009**, 255, 7329-7336. https://doi.org/10.1016/j.apsusc.2009.03.092.
2. Flory, P.J. Statistical mechanics of swelling of network structures. *J. Chem. Phys.* **1950**, *18*, 108-111. https://doi.org/10.1063/1.1747424.
3. Marzocca, A.; Garraza, A.R.; Mansilla, M. Evaluation of the polymer–solvent interaction parameter χ for the system cured polybutadiene rubber and toluene. *Polym. Test.* **2010**, *29*, 119-126. https://doi.org/10.1016/j.polymertesting.2009.09.013.
4. Wang, D.; Tang, Z.; Liu, Y.; Guo, B. Crosslinking diene rubbers by using an inverse vulcanised co-polymer. *Green Chem.* **2020**, *22*, 7337-7342. https://doi.org/10.1039/D0GC02660C.
5. Zhang, X.; Tang, Z.; Guo, B. Regulation of mechanical properties of diene rubber cured by oxa-Michael Reaction via manipulating network structure. *Polymer* **2018**, *144*, 57-64. https://doi.org/10.1016/j.polymer.2018.04.039
